# Preliminary evaluation of a novel group-based motivational interviewing intervention with adolescents: a feasibility study

**DOI:** 10.3389/fpubh.2024.1344286

**Published:** 2024-03-06

**Authors:** Lotte Vallentin-Holbech, Sidsel Helena Karsberg, Anette Søgaard Nielsen, Sarah W. Feldstein Ewing, Kristine Rømer Thomsen

**Affiliations:** ^1^Centre for Alcohol and Drug Research, Aarhus BSS, Aarhus University, Aarhus, Denmark; ^2^Unit for Clinical Alcohol Research, Clinical Institute, University of Southern Denmark, Odense, Denmark; ^3^Department of Psychology, University of Rhode Island, Kingston, RI, United States

**Keywords:** adolescents, motivational interviewing, schools, hazardous alcohol use, health promotion

## Abstract

Drinking is closely intertwined with social life among many adolescents, particularly in Europe. Group-based interventions, such as group-based motivational interviewing (group MI), have shown the capacity to prevent and reduce hazardous drinking and related problems among adolescents, but few examinations have been conducted in a European high school setting. This study examines the preliminary outcomes of a pilot group MI intervention among Danish adolescents. High school students (ages 15–18 years) were randomly allocated to two 1-h group MI sessions delivered in a school setting (*N* = 65) or an assessment only control condition (*N* = 67). Data were collected in August and November 2020 using online self-administrated questionnaires regarding the acceptability of the intervention and past month alcohol use. The pilot group MI intervention showed high feasibility and acceptability in this setting and with this age group. Group MI adolescents significantly reduced peak drinks per drinking day compared to assessment only adolescents (−2.7 drinks, *p* < 0.05). Results are discussed in relation to the metrics being evaluated during COVID-19 lockdown, including increased social restrictions at follow-up compared to baseline. Group MI shows promise for reducing hazardous alcohol use among Danish adolescents. In addition, the findings indicate the importance of building on and extending this work in future larger, better-powered randomized controlled trials.

## 1 Introduction

European adolescents have some of the highest drinking rates throughout the globe across quantity and frequency with the highest drinking rates observed in Denmark, Austria, Germany, Hungary, and the Netherlands (1, 2). Especially in Denmark, the prevalence of heavy drinking is high with 41% of boys and 40% of girls reporting past month intoxication compared to an average of 13% among all of the adolescents from 35 European countries participating in the European School Survey Project on Alcohol and Other Drugs (ESPAD) (1). Like in most other countries, for Danish adolescents, alcohol is closely linked to social life and not perceived by themselves as hazardous (3, 4). Further, heavy drinking has documented serious short-term consequences for adolescents including fighting, injuries, unprotected sex, and unwanted attention on social media, and is linked to long-term harm in adulthood (5–7). These consequences have been shown to interfere with their positive life trajectory, rendering it much more difficult. Thus, reducing adolescent drinking, particularly hazardous drinking, i.e., use that increases the risk or likelihood of negative consequences (e.g., the experience of physical, mental and/or social consequences) (8), is a critical avenue to protect and facilitate adolescent growth and neurodevelopment (4).

An important avenue to prevent adolescents from hazardous alcohol use and risk of related negative consequences is via brief, evidence-based programs that can be implemented in settings where they are already at, such as high schools (9, 10).

Systematic reviews and meta-analyses, to date, highlight that motivational interviewing (MI) is one of the empirical interventions with the strongest support for reducing alcohol use among adolescents (10, 11). MI is a therapeutic approach, which is characterized by being open, strength-based, affirming, non-judgmental, and empathic, with a goal of exploring and resolving ambivalence around the problem behavior (such as hazardous drinking) to support the individual’s intrinsic motivation for behavior change (12). In terms of brief MI (1–2 session), specifically, studies largely conducted within North America show support for group-based MI (group MI) in terms of adolescent alcohol reduction (13, 14). Previous studies have shown that it is essential that the group leaders facilitate change talk (change talk is defined as any self-expressed speech that is an argument for change), roll with individuals’ resistance, support self-efficacy, convey accurate empathy, and facilitate awareness of potential discrepancy between individuals’ current behaviors and short and long-term goals, all in a group setting (12). In terms of fit, treatment studies, predominantly conducted with adults in Europe, reflect that group MI aligns well with European cultures. This alignment is observed in its ability to respect the target culture and effectively decrease adult alcohol use (15–17). Specifically, the open, strength-based, affirming, non-judgmental, and empathic approach within MI (12) has shown acceptability and feasibility when conducted with Danish adults (15, 18).

Despite promising findings from treatment studies in Europe and from prevention studies with adolescents in North America, the degree to which brief group MI is effective as a prevention program aimed specifically at young individuals in a European country such as Denmark, is currently unknown. This is critical to assess, given that European adolescents have some of the highest drinking rates throughout the globe (1, 2).

In this pilot study, we aimed to examine initial outcomes of group MI with Danish high school students (15–18 years). We hypothesized that the group MI would be feasible to implement in a school setting and adolescents would show participation and acceptability. Further, we hypothesized that compared to an assessment only condition, adolescents would decrease past month hazardous alcohol use (measured as peak drink per drinking event) and alcohol-related problems (measured with Rutgers Alcohol Problem Index).

## 2 Methods

### 2.1 Design and procedure

The study is a randomized pilot with two conditions: group MI and assessment only. First-year students at a Danish high school were invited to participate. The school principal helped distribute the written participant information to adolescents and their parents. Adolescents and their parents were informed about the procedure and content of the study and that data were collected for research purposes. The participant information also detailed the participants’ rights including that participation was voluntary, and that they had the right to terminate their participation at any point in time. Participants were further informed that their data would be treated in concordance with the current Danish legislation.

The Central Danish Regional Ethical Committee on Health Research Ethics assessed the study and did not consider the study to qualify as a “health research study”, as defined by the Danish Committee Law § 2. In accordance with the Danish Consolidation Act on Research Ethics Review of Health Research Projects, Consolidation Act number 1083 of 15 September 2017 section 14 (1) the Regional Committee found that the study did not require ethical approval from the Regional Ethical Committees in Denmark (casefile: 1–10–72-148-19). Hence, requirement of ethical approval was waived by the Central Denmark Region Committees on Health Research Ethics.

According to Danish legislation (The Committee Law, the Danish General Data Protection Regulation, and the Danish Data Protection Act), individuals aged 15 or above, can provide independent informed consent for study participation provided that the study only collects information by, e.g., interview or survey, does not involve human biological matter and that participants are not subjected to any interventions involving medicinal products. The current study was approved by and registered at the Danish Data Protection Agency. The study was conducted in accordance with the local legislation and institutional requirements. Further, the ethical standards of the Helsinki declaration were followed, and adolescents completed informed consent prior to participation.

Online surveys were conducted in class during school hours in line with the school administration’s request. The first online survey contained information on the study and a request for informed consent that needed to be read before entering the survey. Once consented, six school classes were randomized (1:1) to receive assessment only or group MI in two 1-h sessions. Specifically, interested, consented adolescents were invited from the class during school hours to a separate room with a research team member who conducted the group MI. Written informed consent was obtained from adolescents randomized to receive group MI prior to the first session. Adolescents in assessment only completed parallel measures at the same time points but received no intervention.

Data were collected in August and November 2020. To measure preliminary intervention effects all participating adolescents completed online surveys 3 weeks before the intervention (baseline) and 2-months post-intervention (follow-up). Among group MI adolescents (only) measures on feasibility were collected 3 weeks post-intervention and audio recordings of MI sessions were used to evaluate MI fidelity (19, 20).

### 2.2 Study sample

**Figure 1:**
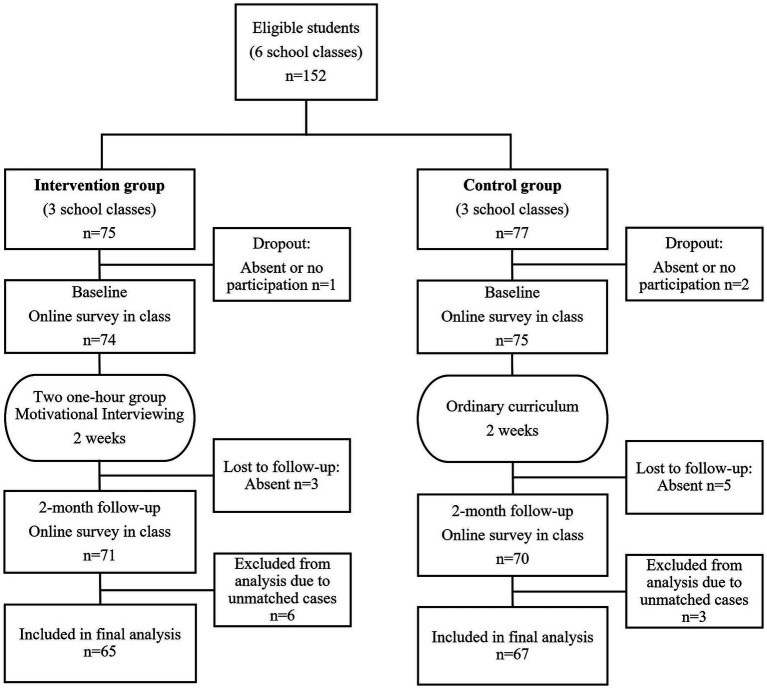
Participant flow through the trial - Danish high school students, 2020.

All first-year students at one Danish high school were invited to participate (*N* = 152 students); *N* = 3 eligible students chose not to participate in the study. In terms of retention, we had complete follow-up data from *N* = 132 students (87%) (see Figure 1). Eight students (5%) did not complete the follow-up survey, and nine students (6%) were excluded from the analyses based on respondent identification codes that could not be located at both baseline and follow-up survey. No significant differences were found in attrition rates for demographics and past 30 days alcohol use at baseline. The sample was 55.3% female and mean age was 16.75 (SD ±0.63) years (see Table 1).

**Table 1:** Characteristics of the study population at baseline – Danish high school students, 2020.

	Group MI *N* = 65	Assessment only *N* = 67	*p*-value
*Gender, % (N)*			
Male	40.00 (26)	44.78 (30)	0.740^b^
Female	56.92 (37)	53.73 (36)	
Other/prefer not to answer	3.08 (2)	1.49 (1)	
*Religion, % (N)*			
Christianity (e.g., Catholic, Protestant)	58.46 (38)	59.70 (40)	0.326^b^
Islam	13.85 (9)	13.43 (9)	
No religion	12.31 (8)	11.94 (8)	
Atheism	7.69 (5)	5.97 (4)	
Other/prefer not to answer	7.69 (5)	8.96 (6)	
Age, mean (SD)	16.75 (0.65)	16.75 (0.61)	0.932^a^
Number of drinking days, past 30 days (TLFB), mean (SD)	4.74 (3.91)	4.64 (5.05)	0.899^a^
Drinks per drinking day, past 30 days (TLFB), mean (SD)	4.00 (3.12)	3.99 (3.16)	0.985^a^
Peak drinks per drinking day, past 30 day (TLFB), mean (SD)	8.35 (5.38)	8.74 (5.32)	0.676^a^
Alcohol-related problems (RAPI, score 0–23), mean (SD)	1.98 (2.57)	1.98 (2.27)	0.499^a^
*Satisfaction with group MI, % (N)*			
Neutral/ Dissatisfied/Very dissatisfied	19.30 (11)		
Satisfied/Very satisfied	80.70 (46)		
Recommend group MI to fellow students, % (N)			
Neutral/Not recommending	21.05 (12)		
Recommending/Highly recommending	78.95 (45)		

^a^*t*-test; ^b^Chi^2^-test. N: number of students; SD: Standard deviation; TLFB: Timeline Followback.

### 2.3 The group MI prevention intervention

The manualized group MI consisted of two 1-h sessions aimed at reducing hazardous drinking and its related risk of negative consequences (21) and was based on group MI interventions developed for adolescents in North America by the fourth author (22). It was adapted to Danish adolescents based on focus group interviews with 26 students (57,7% female, age 16–18 years) at a high school in the same region. Based on data from the focus groups, the main adaptions integrated into the Danish manual for group MI included, but were not limited to; more emphasis on questions regarding “gains from not drinking,” paying more attention to the fact many youth consider use of alcohol as normal at social events and that plays an important role for most, and higher priority (extra time) to the task including value cards (21). Research team members delivered the intervention as group leaders. Adolescents were divided into single-sex groups with 5–7 participants and one group leader facilitating the sessions.

Main themes covered in the first group MI session:

- Tell your story: allowing youth to talk and reflect on their experiences with alcohol (why do they drink? why do they refrain from drinking?).
- Gains from not drinking: allowing youth to explore and reflect on potential benefits for themselves and others.
- Social norms: providing youth with feedback on alcohol use and social norms among first-year students at their school based on the data from the baseline-survey (e.g., “6 out of 10 think it is uncool to get very drunk”), allowing youth to explore and reflect on their own use compared to peer norms.

Main themes covered in the second group MI session:

- Personal values: allowing youth to explore and reflect on what is most important for them now, using and sorting ‘value-cards’ describing 50 different personal values.
- Linking values with behavior: allowing youth to explore and reflect on potential agreement or discrepancy between their personal values and their behavior, including drinking/not drinking.
- Planning/choices regarding alcohol: allowing youth to reflect on how they would like the next party/social gathering in the class/school to be like in terms of alcohol.

### 2.4 Measurements

#### 2.4.1 Feasibility

##### 2.4.1.1 Participation

Group leaders registered number of students attending the two group MI sessions.

##### 2.4.1.2 Acceptability

Participating students indicated how well they liked the group MI and whether they would recommend a fellow student to participate on a 5-point Likert scale from low to high. Further, using open-ended questions the students were asked: “To make the project better, we would like to know what you think about the group sessions; (1) What did you like?, and (2) What could be improved?.” Finally, the students were asked which elements or issues in the group sessions left the biggest impression on them.

##### 2.4.1.3 Fidelity

Similar to a previous study by D’Amico et al. (23), trained independent coders^1^ evaluated MI fidelity using the Motivational Interviewing Treatment Integrity manual version 4.2.1 (MITI 4) (19, 20) based on randomly selected 20-min segments of audio-recorded MI sessions (from 11 out of 26 sessions). Scores measured relational, technical, percentage of complex reflections of all reflections, ratio of reflection to question. Additionally, the independent coders assessed the group leaders’ adherence to the group MI manual.

#### 2.4.2 Outcomes

##### 2.4.2.1 Past month alcohol use

Past month alcohol use was calculated using Timeline Follow back (TLFB) (24). TLFB is a retrospective tool for assessing substance use quantity and frequency patterns using a blank calendar format in which participants can get an overview of dates, weekends, and unique events (e.g., semester start and holidays) over a particular period, e.g., 30 days. It is the gold-standard in self-reported substance use and the online version has similar psychometrics to the in-person TLFB (25, 26). Students were instructed to enter the number of drinks they had day-by-day through the past 30 days. This yielded three past month drinking outcomes: number of drinking days, average drinks per drinking day, and peak drinks per drinking day (representing hazardous drinking).

##### 2.4.2.2 Alcohol-related problems

Alcohol-related problems were measured using Rutgers Alcohol Problem Index (RAPI) among students reporting alcohol use in the past 30 days (27). The index evaluates alcohol problems and consists of 23 items, e.g., “not able to do your homework or study for a test” and “had a fight, argument or bad feeling with a family member.” Each item has response options indicating whether students did or did not experience the specific problem within the past 30 days. All items were summed into a sum score (range 0–23, Cronbach’s alpha = 0.90).

#### 2.4.3 Demographics

Students reported date of birth and age was calculated as years based on the entries in the baseline survey. Further, students reported gender (Male, Female and Other/Prefer not to answer), and religious orientation (Christianity (e.g., Catholic, Protestant), Islam, Not religious, Atheist and Other/Do not know).

### 2.5 Statistical analyses

Analyses were performed using STATA 16.1. Baseline differences between conditions were tested using Pearson’s Chi-square (Chi^2^) test for frequencies and t-test for mean-comparison. Adolescents nested within classes varied from 20 to 26 students and did not significantly differ between conditions (Pearson Chi^2^ (2) = 0.711, *p* = 0.701). Interclass correlation coefficients were all <0.05, indicating that responses from adolescents in the same school class (nest) were not similar. For the three drinking outcomes the skewness and kurtosis test failed to reject the null hypothesis (*p* > 0.05). To evaluate intervention effect on the outcome measures; number of drinking days, drinks per drinking day and peak drinks per drinking day (i.e., hazardous drinking), unadjusted and multilevel mixed-effects linear regression models were fitted. For the outcome measure alcohol-related problems (RAPI23) unadjusted and multilevel negative binomial regression models were used to investigate the effects of the intervention. In all models the follow-up values were included as dependent variable, the baseline score as a predictor, and the intervention condition as an indicator predictor. Multilevel models also included demographic factors (age, gender, and religious orientation), and class (nesting variable) was included as random effect.

A sample size of 50 has been recommended for pilot trials to establish protocol feasibility and obtain reliable variance estimates of primary outcomes for interventions believed to produce a medium effect (28). The current study sample including 132 students will be used to assess the practical meaning of the findings, carefully using the confidence intervals (CI) to interpret the estimates (29).

## 3 Results

### 3.1 Feasibility

In total 80% of group MI adolescents participated in both sessions. Non-attendance was due to the onset of the pandemic (COVID-19 symptoms or related isolation). In total *N* = 74 students were invited to participate in the group MI; *N* = 57 (77%) responded to the survey regarding acceptability with 81% satisfied/very satisfied and 79% recommending/highly recommending the group MI to a fellow student (see Table 1). MITI scores for group MI leaders were rated as fair across all scores, and all group leaders had high (>85%) adherence to the group MI manual.

From the open-ended questions, we found that students in general were happy about; the safe environment provided in the small groups where all had the opportunity to express their opinions, the non-judgmental “atmosphere,” and having time for reflection on drinking and what it entails. E.g., one student wrote: “*I think it was nice to have an open conversation in a small group about it, as it is not something you really talk about otherwise. I also liked that everyone in the group got to say something and be heard.”*

Some students suggested that the MI sessions might be improved if they had less pre-arranged themes/agendas and more to time to talk about other forms for substance use or issues that they found interesting. A few students struggled to feel at ease opening up to their new classmates and actively participate in the discussions during the MI sessions.

The students also elaborated on issues that left the biggest impression on them. Several students wrote that the MI sessions helped them realize that the majority of students do not think that drinking alcohol is needed in order to be accepted by the social group. One student realized “*that there are many who respect that you do not have to drink alcohol at parties”* and another student mentioned that *“I have started to reflect more on how much alcohol I drink and why.”* Additionally, the group MI made them aware of the physical and social impact alcohol has on both the individual, social relations as well as relatives and friends. Several students mentioned a “side-effect” of the MI sessions namely that the sessions made it easier to get to know their new classmates’ opinions and behaviors better.

### 3.2 Drinking outcomes

All surveys were deployed during the pandemic and with increased social restrictions at follow-up compared to baseline (e.g., the assembly ban was reduced from 250 to 10). At follow-up all three drinking measures were significantly reduced in both conditions. In addition, group MI adolescents showed further significant gains in the domain of peak drinks per drinking day (see Table 2).

**Table 2:** Preliminary Outcomes at 2-month follow-up – Danish high school students, 2020.

	2-months follow up		Unadjusted model			Adjusted model			
Drinking outcomes	Group MI Mean (SD)	Assessment only Mean (SD)	Coef.	95% CI	*p*-value	Coef.	95% CI	*p*-value	ICC^c^
Number of drinking days, past 30 days (TLFB)^a^	2.40 (2.77)	2.42 (3.49)	−0.07	−0.82; 0.69	0.353	−0.02	−0.75; 0.72	0.406	0.003
Drinks per drinking day, past 30 days (TLFB)^a^	3.13 (3.07)	3.72 (4.32)	−0.60	−1.67; 0.48	0.266	−0.58	−1.61; 0.45	0.266	<0.001
Peak drinks per drinking day, past 30 days (TLFB)^a^	5.93 (3.88)	8.09 (7.94)	**−2.69**	**−5.12; −0.26**	**0.030**	**−2.70**	**−5.06; −0.33**	**0.026**	<0.001
Alcohol-related problems (RAPI, score 0–23)^b^	1.30 (2.52)	1.11 (2.22)	0.36	−0.37; 1.09	0.331	0.14	−0.55; 0.84	0.685	na

Unadjusted models include baseline values. Adjusted models include baseline values, age, gender and religion, and school class is included as random effect.

a Estimates based on linear regression models with corresponding 95% confidence interval (CI) and *p*-value.

b Estimates based on negative binomial regression models with corresponding 95% CI and *p*-value.

c Residual intraclass correlation between classes. na: analysis not applicable. Significant effect estimates are shown in bold.

## 4 Discussion

The study aimed to assess group MI as a prevention intervention with Danish adolescents (15–18 years) in a school setting. Findings reflect feasibility in terms of implementation in a Danish school setting, even in the midst of a pandemic, and show promise for reducing hazardous drinking among adolescents. Specifically, the group MI and related assessments were delivered during the pandemic. However, even in this context, the group MI and surveys were successfully integrated into the school day, with high participation and acceptability by students and administrators. Moreover, the open-ended questions showed that students enjoyed participating in the MI sessions. They found the topics discussed appealing and made them reflect about their own drinking habits. This is consistent with previous studies which have suggested that high appeal of the elements in prevention interventions could increase students’ attention to the intervention content and messages and in turn help them to comprehend and reflect on the key elements in the MI sessions (30, 31).

Given that alcohol use is highly integrated into adolescents’ social activities, it is not a surprise that we found significant reductions in drinking and related consequences in both conditions at follow-up, where COVID-19 restrictions had been increased (compared to baseline) and hence adolescents were largely in pandemic-related isolation. Considering the strong link between social life/events and alcohol use among adolescents in this region of the world, the COVID-19 restrictions enforced at follow-up (e.g., the assembly ban was constricted from 250 persons in August to 10 persons in November (32)) very likely were related to the decreased alcohol use, as observed in other European studies during this period where governments enforced restrictions due to the COVID-19 (33–35). Studies have shown that the pandemic caused a general concern in the population and that the related consequences of the pandemic contributed to increased alcohol use among adults (36). However, adolescents did not increase their use and in a study of 352 first-year students from three high schools in Denmark, we found a marked decrease across similar measures of past month alcohol use and related negative consequences from August 2020 to November 2020 (35).

Notably, group MI adolescents showed significantly greater reductions in peak drinks per drinking day compared to the assessment only condition. This indicates that this group MI intervention has the potential for reducing hazardous alcohol use. Hazardous alcohol use increases the risk of negative consequences including the experience of physical, mental and/or social consequences. The fact that we did not see larger reductions in negative consequences (RAPI) may be related to the short timeframe of the follow-up or the increased COVID-19 restrictions at follow-up that restricted social life in both groups. Overall, the results are consistent with previous studies, in North America and other parts of the globe, that reflect that group MI alcohol prevention programs can reduce adolescent substance use (13, 14). In the study by D’Amico and colleagues (14) they found that group MI reduced alcohol use and related consequences, and created a more satisfying experience for North American teens, compared to a usual care condition.

Some challenges across prevention programs include stigma surrounding treatment-seeking in this age group (37). Additionally, hazardous drinking is closely interwoven into family and social culture in many European countries (2, 37), and many adolescents do not see heavy drinking as a behavior that may lead to harm (3–5). These issues may seem benign but feed into one of the most pressing public health concerns; that adolescents will make risky choices while intoxicated resulting in very serious consequences (e.g., head injury via traffic accident; a victim of serious sexual assault or rape; making a foolish mistake that costs them life or limb). Additionally, delivering the intervention to all students in a class (as opposed to singling out students with hazardous or at-risk use) might avoid the stigmatization of adolescents who are engaging in hazardous drinking, and it offers maximal reach for the range of adolescent drinking, from adolescents contemplating initiating drinking to adolescents increasing their drinking, to adolescents engaged in hazardous use. Furthermore, previous studies have shown that integrating MI interventions into adolescents’ existing environment (e.g., during the school day), is a highly effective way to bolster participation (28, 38). Further, studies have proven that interventions conducted in a group setting are highly relevant for this age group. This is likely due to the developmental significance of incorporating peer communities within the intervention (13, 38).

In summary, if we are to prevent adolescents from entering into hazardous alcohol use and related consequences, we must find brief, effective interventions that can be implemented where the adolescents already are (e.g., schools), and that do not require the adolescents to have a high perception of harm. Interventions with a socio-ecological approach that emphasize the structural context while incorporating the individual, social (parents and peers), and psychological influences might hold the most promise for adolescents at-risk, or transitioning-into-risk (4, 39–41). Hence, future studies should examine how group MI may intersect with structural interventions (e.g., targeting school alcohol policies). Findings indicate the importance of building on and extending this work in larger, better-powered studies in a true randomized controlled trial with an active comparison condition post-pandemic, including longer-term follow-up.

Some limitations of this study should be noted. Data were obtained from self-administered questionnaires and therefore over-and underreporting cannot be ruled out. However, the online survey allowed adolescents to answer sensitive questions in an anonymous manner (42). Only one follow-up survey restricts the results to short-term effects. Nevertheless, the 2-month follow-up survey made it possible to measure feasibility and initial effects without major disruptions to the school curriculum. The strength of our design is that many factors (at the school) were kept constant, however, we cannot rule out potential ‘spillover effects’ between the two conditions. Lastly, the fact that the study was conducted during the COVID-19 pandemic limits generalizability of the findings. At the same time, the finding that group MI impacted our measure of hazardous drinking (compared to assessment only), despite the fact that Danish adolescents generally decreased drinking in this period because of increased restrictions (at follow-up) that limited social life, could also be interpreted as providing additional support to group MI.

## 5 Conclusion

Group MI is highly feasible as a prevention intervention and shows promise for reducing hazardous drinking among Danish adolescents in high school, particularly in the domain of peak drinks per drinking day.

## Data availability statement

The raw data supporting the conclusions of this article will be made available by the authors, without undue reservation.

## Ethics statement

Ethical approval was not required for the study involving humans in accordance with the local legislation and institutional requirements. The studies were conducted in accordance with the local legislation and institutional requirements. The participants provided their written informed consent to participate in this study.

## Author contributions

LV-H: Formal analysis, Investigation, Methodology, Project administration, Writing – original draft, Writing – review & editing. SK: Investigation, Writing – review & editing. AN: Validation, Writing – review & editing. SF: Conceptualization, Funding acquisition, Methodology, Writing – review & editing. KT: Conceptualization, Funding acquisition, Investigation, Methodology, Supervision, Writing – review & editing.

## Footnotes

^1^https://app-kodningslab.cloud.sdu.dk/

## References

[1] ESPAD Group. ESPAD Report. Results from the European school survey project on alcohol and other drugs. Luxembourg: EMCDDA Joint Publications, Publications Office of the European Union (2019).

[2] WHO. Global status report on alcohol and health 2018. Geneva: World Health Organization (2018).

[3] Christensen ASP Behrens CL Hansen L Zachariasen E. Unges alkoholvaner i Danmark 2017 - en kortlægning [Youths alcohol habits in Denmark 2017 - a survey]. Copenhagen: Kræftens Bekæmpelse og TrygFonden smba (2018).

[4] Silvers JA Squeglia LM Rømer Thomsen K Hudson KA Feldstein Ewing SW. Hunting for what works: adolescents in addiction treatment. Alcohol Clin Exp Res. (2019) 43:578–92. doi: 10.1111/acer.13984, PMID: 30779445 PMC6443447

[5] Meyer MKH Lundgaard PB Zachariasen E Christensen ASP. Unges alkoholvaner i Danmark 2019 [youths alcohol habits in Denmark 2019]. Copenhagen: Kræftens Bekæmpelse og TrygFonden smba (2020).

[6] Morales AM Jones SA Kliamovich D Harman G Nagel BJ. Identifying early risk factors for addiction later in life: a review of prospective longitudinal studies. Curr Addict Rep. (2020) 7:89–98. doi: 10.1007/s40429-019-00282-y, PMID: 33344103 PMC7747788

[7] Ranganath P Hjetland GJ Finserås TR Brunborg GS Hesse M Skogen JC. Negative experiences, social exclusion and unwanted attention on social media: exploring the association with adolescent alcohol use. BMC Public Health. (2022) 22:2361. doi: 10.1186/s12889-022-14679-4, PMID: 36527010 PMC9756586

[8] Saitz R Miller SC Fiellin DA Rosenthal RN. Recommended use of terminology in addiction medicine. J Addict Med. (2021) 15:3–7. doi: 10.1097/ADM.0000000000000673, PMID: 32482955

[9] Hodder RK Freund M Wolfenden L Bowman J Nepal S Dray J. Systematic review of universal school-based 'resilience' interventions targeting adolescent tobacco, alcohol or illicit substance use: a meta-analysis. Prev Med. (2017) 100:248–68. doi: 10.1016/j.ypmed.2017.04.003 28390835

[10] Tinner L Palmer JC Lloyd EC Caldwell DM Macarthur GJ Dias K. Individual-, family- and school-based interventions to prevent multiple risk behaviours relating to alcohol, tobacco and drug use in young people aged 8-25 years: a systematic review and meta-analysis. BMC Public Health. (2022) 22:1111. doi: 10.1186/s12889-022-13072-5, PMID: 35658920 PMC9165543

[11] Steele DW Becker SJ Danko KJ Balk EM Adam GP Saldanha IJ. Brief behavioral interventions for substance use in adolescents: a Meta-analysis. Pediatrics. (2020) 146:e20200351. doi: 10.1542/peds.2020-0351, PMID: 32928988

[12] Miller WR Rollnick S. Motivational interviewing: helping people change and grow. 4th ed. New York, NY: The Guilford Press (2023).

[13] Martín-Pérez C Navas JF Perales JC López-Martín Á Cordovilla-Guardia S Portillo M. Brief group-delivered motivational interviewing is equally effective as brief group-delivered cognitive-behavioral therapy at reducing alcohol use in risky college drinkers. PLoS One. (2019) 14:e0226271. doi: 10.1371/journal.pone.0226271, PMID: 31821350 PMC6903743

[14] D'Amico EJ Hunter SB Miles JN Ewing BA Osilla KC. A randomized controlled trial of a group motivational interviewing intervention for adolescents with a first time alcohol or drug offense. J Subst Abus Treat. (2013) 45:400–8. doi: 10.1016/j.jsat.2013.06.005, PMID: 23891459 PMC3826597

[15] Andersen K Behrendt S Bilberg R Bogenschutz MP Braun B Buehringer G. Evaluation of adding the community reinforcement approach to motivational enhancement therapy for adults aged 60 years and older with DSM-5 alcohol use disorder: a randomized controlled trial. Addiction. (2020) 115:69–81. doi: 10.1111/add.14795, PMID: 31454444

[16] Gaume J Bertholet N McCambridge J Magill M Adam A Hugli O. Effect of a novel brief motivational intervention for alcohol-intoxicated young adults in the emergency department. JAMA Netw Open. (2022) 5:e2237563. doi: 10.1001/jamanetworkopen.2022.37563, PMID: 36269355 PMC9587483

[17] Nadkarni A Massazza A Guda R Fernandes LT Garg A Jolly M. Common strategies in empirically supported psychological interventions for alcohol use disorders: a meta-review. Drug Alcohol Rev. (2022) 42:94–04. doi: 10.1111/dar.13550 36134481 PMC10087716

[18] Kramer Schmidt L Moyers TB Nielsen AS Andersen K. Is fidelity to motivational interviewing associated with alcohol outcomes in treatment-seeking 60+ year-old citizens? J Subst Abus Treat. (2019) 101:1–11. doi: 10.1016/j.jsat.2019.03.004, PMID: 31174708

[19] Kramer Schmidt L Andersen K Nielsen AS Moyers TB. Lessons learned from measuring fidelity with the motivational interviewing treatment integrity code (MITI 4). J Subst Abus Treat. (2019) 97:59–67. doi: 10.1016/j.jsat.2018.11.004, PMID: 30577900

[20] Moyers TB Rowell LN Manuel JK Ernst D Houck JM. The motivational interviewing treatment integrity code (MITI 4): rationale, preliminary reliability and validity. J Subst Abus Treat. (2016) 65:36–42. doi: 10.1016/j.jsat.2016.01.001, PMID: 26874558 PMC5539964

[21] Vallentin-Holbech L Karsberg SH Søgaard Nielsen A Feldstein Ewing SW Rømer Thomsen K. Manual: Dit valg! Sundhedsfremme blandt danske gymnasieelver. [Treatment Manual for Group-based MI among Youth: “Your Choice! Health Promotion among Danish Students in High School”]. Aarhus, Denmark: Center for Alcohol and Drug Research, Aarhus University. (2020)., PMID:

[22] Feldstein Ewing AW. The Adolescent Substance Use Check-Up: Intervention Manual for Project MINA. Portland, Oregon: Oregon Health & Science University. (2017)., PMID:

[23] D'Amico EJ Osilla KC Miles JNV Ewing B Sullivan K Katz K. Assessing motivational interviewing integrity for group interventions with adolescents. Psychol Addict Behav. (2012) 26:994–1000. doi: 10.1037/a0027987, PMID: 22642853 PMC3540190

[24] Sobell LC Cunningham JA Sobell MB. Recovery from alcohol problems with and without treatment: prevalence in two population surveys. Am J Public Health. (1996) 86:966–72. doi: 10.2105/AJPH.86.7.966, PMID: 8669520 PMC1380437

[25] Hareskov Jensen N Vallentin-Holbech L Dash G Feldstein Ewing SW Rømer TK. Validity of an online, self-administered timeline Followback for alcohol use with adolescents. Front Psych. (2023) 14:1221487. doi: 10.3389/fpsyt.2023.1221487, PMID: 38098631 PMC10720705

[26] Martin-Willett R Helmuth T Abraha M Bryan AD Hitchcock L Lee K. Validation of a multisubstance online timeline Followback assessment. Brain Behav. (2020) 10:e01486. doi: 10.1002/brb3.1486, PMID: 31793226 PMC6955818

[27] White HR Labouvie EW. Towards the assessment of adolescent problem drinking. J Stud Alcohol. (1989) 50:30–7. doi: 10.15288/jsa.1989.50.30 2927120

[28] Bryan AD Magnan RE Gillman AS Yeater EA Feldstein Ewing SW Kong AS. Effect of including alcohol and Cannabis content in a sexual risk-reduction intervention on the incidence of sexually transmitted infections in adolescents. JAMA Pediatr. (2018) 172:e175621. doi: 10.1001/jamapediatrics.2017.5621, PMID: 29435591 PMC5875326

[29] Dziak JJ Dierker LC Abar B. The interpretation of statistical power after the data have been gathered. Curr Psychol. (2020) 39:870–7. doi: 10.1007/s12144-018-0018-1, PMID: 32523323 PMC7286546

[30] Domitrovich CE Bradshaw CP Poduska JM Hoagwood K Buckley JA Olin S. Maximizing the implementation quality of evidence-based preventive interventions in schools: a conceptual framework. Adv Sch Ment Health Promot. (2008) 1:6–28. doi: 10.1080/1754730X.2008.9715730, PMID: 27182282 PMC4865398

[31] Vallentin-Holbech L Rasmussen BM Stock C. Does level of received intervention dose have an impact on the effectiveness of the social norms alcohol prevention program the GOOD life? Front Public Health. (2019) 7:245. doi: 10.3389/fpubh.2019.00245 31555631 PMC6722864

[32] Danish Health Authority. COVID-19 Risikovurdering, strategi og tiltag ved epidemi i Danmark [COVID-19 Risk assessment, strategy and restrictions in Denmark]. Copenhagen: Danish Health Authority (2020).

[33] Bollen Z Pabst A Creupelandt C Fontesse S Lannoy S Pinon N. Prior drinking motives predict alcohol consumption during the COVID-19 lockdown: a cross-sectional online survey among Belgian college students. Addict Behav. (2021) 115:106772. doi: 10.1016/j.addbeh.2020.106772, PMID: 33418433

[34] Evans S Alkan E Bhangoo JK Tenenbaum H Ng-Knight T. Effects of the COVID-19 lockdown on mental health, wellbeing, sleep, and alcohol use in a UK student sample. Psychiatry Res. (2021) 298:113819. doi: 10.1016/j.psychres.2021.113819, PMID: 33640864 PMC9754711

[35] Vallentin-Holbech L Feldstein Ewing SW Rømer TK. Hazardous alcohol use among Danish adolescents during the second wave of COVID-19: Link between alcohol use and social life. Nordic studies on alcohol and drugs. (2023). Open Access. 10.1177/14550725221149489 PMC10101166 37063817

[36] Kilian C Rehm J Allebeck P Braddick F Gual A Barták M. Alcohol consumption during the COVID-19 pandemic in Europe: a large-scale cross-sectional study in 21 countries. Addiction. (2021) 116:3369–80. doi: 10.1111/add.15530 34109685

[37] Cousijn J Luijten M Feldstein Ewing SW. Adolescent resilience to addiction: a social plasticity hypothesis. Lancet Child Adolesc. (2018) 2:69–78. doi: 10.1016/S2352-4642(17)30148-7 PMC6373770 30169197

[38] Feldstein Ewing SW Walters S Baer J. Group motivational interviewing with adolescents and young adults: strengthening the developmental fit In: Wagner CC Ingersoll KS, editors. Group motivational interviewing. New York, NY: The Guilford Press (2012).

[39] DiClemente CC Corno CM Graydon MM Wiprovnick AE Knoblach DJ. Motivational interviewing, enhancement, and brief interventions over the last decade: a review of reviews of efficacy and effectiveness. Psychol Addict Behav. (2017) 31:862–87. doi: 10.1037/adb0000318, PMID: 29199843

[40] Self KJ Borsari B Ladd BO Nicolas G Gibson CJ Jackson K. Cultural adaptations of motivational interviewing: a systematic review. Psychol Serv. (2022) 20:7–18. doi: 10.1037/ser0000619 35130010 PMC10161132

[41] Rømer Thomsen K Vallentin-Holbech L Xylander S Wellnitz KB Tolstrup J Nielsen AS. Prevention of hazardous use of alcohol among high school students: a study protocol for the randomized controlled trial ‘our choice’. BMC Public Health. (2023) 23:2079. doi: 10.1186/s12889-023-16976-y, PMID: 37875851 PMC10594784

[42] Ekholm O Strandberg-Larsen K Christensen K Grønbæk M. Comparison of assessment methods for self-reported alcohol consumption in health interview surveys. Eur J Clin Nutr. (2008) 62:286–91. doi: 10.1038/sj.ejcn.1602728, PMID: 17375115

